# Comparison of intralesional 5-fluorouracil versus intralesional triamcinolone acetonide for keloid treatment: A prospective randomised double-blind controlled study

**DOI:** 10.6026/973206300221244

**Published:** 2026-02-28

**Authors:** Vishwa Deep, Prabhat Shrivastava

**Affiliations:** 1 Department of Burns and Plastic Surgery, Lok Nayak Hospital and associated Maulana Azad Medical College, New Delhi-110002, India

**Keywords:** Keloid, 5-fluorouracil, triamcinolone acetonide, intralesional injection, Vancouver scar scale, wound healing

## Abstract

Keloid scars pose significant treatment challenges due to excessive collagen deposition, causing pain, functional impairment and psychological distress despite multiple available interventions. Therefore, it is of interest to compare intralesional 5-fluorouracil (5-FU, 50 mg/mL) versus triamcinolone acetonide (40 mg/mL) in 30 patients randomly assigned to two equal groups. Vancouver Scar Scale parameters (height, pliability, vascularity, pigmentation), pain (Visual Analogue Scale) and pruritus were assessed at treatment end and 2-month follow-up. Both treatments achieved significant VSS improvement (p<0.001), with 93.3% 5-FU and 100% triamcinolone patients reaching total VSS ≤5; 5-FU showed faster response (40% endpoint by session 5 vs 20%) and fewer adverse reactions. Intralesional 5-FU emerges as a superior, less toxic alternative to triamcinolone for keloid management, offering equivalent efficacy with quicker clinical response and better safety profile.

## Background:

Keloids are benign fibro proliferative diseases that develop as a result of an abnormal wound healing reaction to an excess and chaotic collagen deposition that is beyond the usual limits of the wound injury [1]. These pathological scars occur as a result of excess proliferation of fibroblasts and uncontrolled accumulation of the extracellular matrix, leading to elevated, indurated, erythematous lesions, which can generate physical discomfort, impairment of functions and severe psychological misery [2]. The name of these lesions, the cheloid, named after the Greek crab claw, was first applied in the early nineteenth century, to denote the lateral extensions of these lesions into normal tissue [3]. Epidemiology of the keloid formation proves to be very different in dissimilar populations, making the darkly pigmented individuals the most vulnerable, with up to 15 time's higher susceptibility than individuals with lighter skin [4]. The disorder is mostly experienced in people who are 10-30 years old and who anatomically prefer high skin tension areas such as the presternal area, the deltoid area, the upper back and the ear lobes [5]. Different precipitating factors have been distinguished, such as surgical procedures, traumatic injuries, burns, inflammatory conditions of the skin and immunisation sites, but spontaneous development with no identifiable cause is present in a significant percentage of cases [6]. Pathophysiology of Keloid formation is a complicated dysregulation of the normal wound healing cascade and keloid-derived fibroblasts have been shown to have up to twenty-fold higher rates of collagen synthesis than normal dermal fibroblasts [7]. This hyperplastic fibroblastic activity causes deposition of thick hyalinized bundles of collagen in disorganised whorls, distinguishing keloid histologically from normal scars, where collagen fibres are laid parallel to the skin surface [8].

The knowledge of such molecular processes has been used to develop treatment approaches for fibroblast and collagen regulation. Although many treatment modalities such as surgical excision, radiation therapy, cryotherapy, laser treatment, pressure therapy, silicone gel application and various intralesional pharmacological agents exist, none of them have shown a high degree of consistency and recurrence rates are still pathetic [9]. The primary mode of keloid therapy during the past few decades has been intralesional corticosteroid injections, especially of triamcinolone acetonide, where the success stories have reported between 50 and 100 per cent success [10]. Nonetheless, corticosteroid treatment has severe side effects such as skin atrophy, telangiectasias and hypopigmentation and recurrence rates ranging between 9-50 per cent [11]. The identification of similarity between keloid behaviour and the patterns of neoplastic growth has led to the exploration of antimitotic chemotherapeutic drugs as alternative therapeutic options [13]. 5-Fluorouracil (5-FU) is a pyrimidine analogue that inhibits the thymidylate synthase and, consequently, interferes with DNA synthesis as an effective treatment modality as a result of its ability to suppress rapidly proliferating fibroblasts and promote scar degradation [13]. The clinical studies have shown variable success rates between 45 and 96% with intralesional application of 5-FU, with inconsistent outcome measures and follow-up durations [14]. In spite of the increasing interest in the use of 5-FU in the treatment of keloid, there is still no agreement as to its relative efficacy with triamcinolone acetonide, optimal dosage regimens, or even relative safety profiles [15]. The literature has been described with methodological limitations such as suboptimal study designs, lack of blinding, use of variable outcome evaluation instruments and lack of follow-ups, within which the ability to assess recurrence rate is inadequate [16]. Also, there is limited direct comparison of these two modes of therapy using validated, standardised instruments of scar assessment. Therefore, it is of interest to compare the intralesional 5-fluorouracil and intralesional triamcinolone acetonide as far as their effectiveness in the treatment of keloid scars, comparing the validated Vancouver Scar Scale and to compare the comparative safety features and incidence of adverse effects of the two therapeutic modalities.

## Materials and Methods:

## Study design and setting:

The study was a prospective, randomised, controlled and blinded interventional study conducted at the Department of Burns and Plastic Surgery in a tertiary care teaching hospital in New Delhi, India, in 14 months (between July 2019 and September 2020). The protocol of the study was approved by the Institutional Ethics Committee before the study was started. The ethical principles in the Declaration of Helsinki on research on humans were followed in all the procedures undertaken.

## Population and sample size of the study:

The research population included those patients who walked into the outpatient department and were clinically diagnosed with keloid scars. The calculation of sample size was done under the use of standard statistical formulae using some previous literature, with a power of 80 and 95 as the level of significance. As a result of practical limitations such as time constraints of the study and availability of patients, a sample of convenience was used, consisting of 30 patients, with 15 patients in each treatment group.

## Inclusion and Exclusion criteria:

The study was restricted to patients between the age between 18 and 60 years who have keloid scars for a period of more than six months and scar size not exceeding 20 cm^2^. In cases where the patients came with multiple keloid lesions, two scar sites were considered as the maximum. The exclusion criteria included pregnancy and lactation, comorbidities, such as diabetes mellitus, high blood pressure, coronary artery disease, chronic liver disease and intralesional steroid injection within the last six months and the presence of active infection in the keloid lesion.

## Randomisation and blinding:

An English random number was used to assign enrolled patients to 2 groups, Group F (5-FU group) and Group T (triamcinolone group), which consisted of 15 patients each. The study was done under a state of double-blinding, whereby the patients and the independent assessor were not aware of the allocation of treatment. The only person who was aware of the drug used was the investigator who made the injections.

## Treatment protocol:

Group F patients were treated with an intralesional injection of 5-fluorouracil at a concentration of 50 mg/mL at a dose of 0.2 mL/cm 2 (10mg/cm^2^) and a maximum of 100mg/session. Group T patients were treated with intralesional triamcinolone acetonide at 40 mg/mL and a dosage of 0.1 mL/cm^2^ (4mg/cm^2^) with an upper limit of 80 mg/kg at one session. Three weeks after every injection was done with sterile disposable 1 mL insulin syringes with 26-gauge needles, with not more than eight sessions or until significant improvement was realised, which was determined as a total Vancouver Scar Scale score of 0-5. The method of injection was through direct intralesional injection at a sustained pressure till the lesion blanched. Larger lesions were treated with multiple sites of 1 cm distance so that the whole keloid mass could be homogeneously covered with the drug. The study drugs did not contain any local anaesthetic or were not used before injection.

## Outcome assessment:

Clinical evaluation was conducted with a senior experienced independent observer who was blinded at baseline, at each treatment session and every two months after the therapy to evaluate the clinical assessment. Vancouver Scar Scale (VSS) was the main outcome measure and it assesses four variables, namely: height (measured using Vernier callipers), pliability (palpation), vascularity (scored after blanching and comparison with surrounding skin) and pigmentation (scored by visual observation). Secondary outcome measures were the pain measurement utilising the Visual Analogue Scale (VAS) of 0 to 10, as well as the itch score on a four-point scale (0: no itch; 1: mild itch; 2: moderate itch; 3: severe itch). The negative results, such as skin ulceration, atrophy, telangiectasia and infection, were recorded during the visit. All patients had their photographs captured at every session.

## Statistical analysis:

The data were also keyed into Microsoft Excel and they were analysed via the SPSS version 25.0 program. Mean standard deviation was used to state the quantitative variables with normal distribution and those that are not normally distributed median and interquartile range. Student t-test, used to compare normally distributed data and the Mann-Whitney U test, used to compare non-parametric data, were used in between-group comparisons. The Friedman test was applied to within-group comparisons over time on more than two occasions. It was ensured that a 95 per cent confidence interval was applied and that the p-values below 0.05 were taken as statistically significant ones.

## Results:

Complete data were available for all 30 enrolled patients, with no dropouts recorded during the study period. The demographic and clinical characteristics of both treatment groups are presented in Table 1. The mean age of patients was 32.6 years overall, with comparable distribution between Group F (33.2±12.4 years) and Group T (32.0±11.8 years; p = 0.833). Male patients predominated in both groups, with an overall male-to-female ratio of 2:1. The mean duration of keloid lesions was 3.0±1.46 years in Group F and 2.4±1.24 years in Group T (p = 0.236). The presternal region was the most common anatomical site, accounting for 53.3% of all lesions, followed by the deltoid region (20%) and face (13.3%). Idiopathic aetiology was identified in 36.67% of cases, followed by surgical scars (23.33%), infection (20%) and trauma (20%). No statistically significant differences were observed between groups for any baseline characteristic. The two treatment modalities showed statistically significant improvement on all parameters of VSS except pigmentation during the study period. Table 2 shows the change in the VSS scores in treatment sessions in detail. There was a significant reduction in mean keloid height at baseline and final follow-up in both groups. Mean height in Group F decreased from 0.27±0.07 cm in session 1 to 0.03±0.03 cm in follow-up 2 (p < 0.001). Equally, Group T showed the shortening of 0.32±0.17 cm to 0.05±0.05 cm (p < 0.001). Full flattening occurred in 3 (20 per cent) patients in Group F and 4 (26.7 per cent) patients in Group T. No statistically significant difference in groups at various sessions was noted, as both groups had excellent improvement in height (76-100 per cent). Both groups showed significant improvements in the pliability scores (p < 0.001). The mean pliability score reduced to 2.67 + 0.62 and then 1.20 + 0.41 in Group F and Group T, respectively. The follow-up period had a statistically significant enhancement between groups (p = 0.013). The scores of vascularity showed a progressive decrease in each group and the final assessment of each group showed statistically significant improvement compared to baseline (p < 0.001). Group F improved in session 2 and Group T improved in session 3, but there was no significant difference between groups. There was no significant change in both within-group and between-group comparison of pigmentation scores in both treatment modalities over the period of the study (p > 0.05). Progressive improvement in total VSS score was observed in both treatment groups throughout the study period (p < 0.001). Notably, 40% (6/15) of patients in Group F achieved the therapeutic endpoint (total VSS score ≤5) by session 5, compared to only 20% (3/15) in Group T, although this difference did not reach statistical significance (p = 0.233). By the conclusion of the study, 93.3% (14/15) of patients in Group F and 100% (15/15) of patients in Group T achieved significant improvement. Pain and pruritus demonstrated significant improvement in both groups. The proportion of patients experiencing pain decreased from 66.7% to 20% in Group F and from 80% to 26.7% in Group T at the end of therapy (p < 0.001 for both). Complete resolution of pruritus was achieved by session 7 in all patients of both groups, even in those with residual scar elevation. The incidence and distribution of adverse effects are summarised in Table 3. Overall, adverse effects were observed in 3 patients (20%) in Group F compared to 6 patients (40%) in Group T, although this difference did not reach statistical significance (p = 0.232). Superficial ulceration was the only adverse effect observed in Group F (20%), which healed within 5-7 days with topical antibiotic application. In Group T, telangiectasia was the most common adverse effect (33.3%), followed by skin atrophy (20%) and ulceration (6.67%). The incidence of telangiectasia and atrophy was significantly higher in Group T compared to Group F (p = 0.007 and p = 0.033, respectively). No clinical evidence of recurrence was observed in any patient in either treatment group during the follow-up period extending to two months after completion of the eighth treatment session.

## Discussion:

These results of the prospective randomised controlled trial show that the two intralesional 5-fluorouracil and triamcinolone acetonide are effective treatment modalities in the treatment of keloid, with equivalent overall efficacy but significant dissimilarity in the characteristics of response to treatment and the circumstances of adverse effects. The role of both methods in clinical practice is confirmed by significant change in the parameters of the Vancouver Scar Scale, such as height, pliability and vascularity, which showed significant improvement after treatment with either of the two agents. The similarity in the effectiveness of 5-FU and triamcinolone acetonide in the current research is consistent with the past comparative studies that found similar efficacy in treatment with these two modalities [17]. The triamcinolone mechanism of action comprises the inhibition of inflammatory cell migration, vasoconstriction and disruption of nutrient supply to the wound and antimitotic effects on fibroblasts and keratinocytes, whereas 5- FU mechanism of action is the inhibition of thymidine synthase, which results in the disruption of deoxyribonucleic acid synthesis by rapidly proliferating fibroblasts [18]. An interesting result of the study was the achieved better rate of therapeutic endpoints when using 5-FU as the therapy, though 40 per cent of patients in the 5-FU group had achieved significant improvement by the fifth session, compared to 20 per cent of the triamcinolone group. This finding indicates that 5-FU could have some benefits in terms of treatment regimen and patient comfort, but both of the agents ended up with similar results at the end of the treatment period. Past studies have also indicated inconsistent results on the relative rate of therapeutic response and the results of some studies indicated that 5-FU was more effective at producing height reduction more quickly than the treatment [16]. A considerable reduction in the incidence of telangiectasia and skin atrophy with 5-FU over triamcinolone is a clinically significant benefit, especially in keloids in cosmetically sensitive regions. The side effects of intralesional corticosteroids, such as skin atrophy, telangiectases and pigment, are always reported in the literature and are a great drawback of this treatment method [10]. Although 5-FU may lead to superficial ulceration, the cytotoxic effect of this drug does not seem to induce the dermal atrophy and vascular alteration of corticosteroids. This resolution of pruritus, which was demonstrated in both treatment groups by week seven, including patients with residual scar elevation, indicates that improved symptoms may be one of the initial stages before morphological alterations. The practicality of the finding in patient counselling and treatment expectations is that control of distressing symptoms can be attained comparatively at the beginning of the therapeutic process [19]. The methodological strength of the study is the use of the validated Vancouver Scar Scale in the measurement of outcomes, as this is a limitation of the previous studies in this area because the majority of them have used heterogeneous measures of outcomes [20]. Total VSS scores calculated in each session gave an objective assessment of treatment response and allowed for direct comparison between treatment modalities. The lack of recurrence on the follow-up period is promising but should be taken with caution due to the rather short duration of observation, two months after the treatment. In the past, recurrent rates have been documented at one year at 33% and 50 years at five years after corticosteroid therapy, which highlights the necessity of a long-term follow-up to adequately evaluate the long-term treatment outcomes [20]. There are a number of limitations of this study that should be mentioned. The sample size is relatively small, which restricts statistical power to identify smaller effect sizes and makes it impossible to make conclusive decisions on treatment protocols. Although the follow-up period is sufficient in determining the response to treatment in the short-term, it is inadequate to determine long-term recurrence rates. Also, the single-centre design can be a limitation to the generalizability to other populations and clinical settings. The results of the present research can be considered as adding to the existing body of evidence that supports the use of 5-FU as a potential alternative in the treatment of keloid as an alternative to triamcinolone acetonide. The similar efficacy, quicker therapeutic response and more desirable side effect profile noted with 5-FU are indicative of the fact that the agent does deserve consideration as a first-line therapy agent, especially in patients with reservations against corticosteroid- related adverse effects or those who have keloids in cosmetically prominent areas.

## Conclusion:

We show that intralesional 5-fluorouracil and triamcinolone acetonide achieve equivalent Vancouver Scar Scale improvements in keloid management. 5-FU demonstrated faster response rates (greater mid-treatment improvement) and a superior safety profile without telangiectasia or skin atrophy seen in triamcinolone patients. Intralesional 5-FU emerges as the preferred keloid treatment due to comparable efficacy, quicker clinical response and fewer adverse events, warranting larger multicenter validation.

## Figures and Tables

**Table 1:** Baseline demographic and clinical characteristics of study participants

**Parameter**	**Group F (n=15)**	**Group T (n=15)**	**p-value**
Age (years), Mean±SD	33.2±12.4	32.0±11.8	0.833
Sex (Male/Female)	4-Nov	6-Sep	0.439
Keloid duration (years), Mean±SD	3.0±1.46	2.4±1.24	0.236
Anatomical Site, n (%)			>0.05
Presternal	8 (53.3%)	8 (53.3%)	
Deltoid	2 (13.3%)	4 (26.7%)	
Face	4 (26.7%)	0 (0%)	
Other sites	1 (6.7%)	3 (20%)	
Aetiology, n (%)			0.563
Idiopathic	6 (40%)	5 (33.3%)	
Surgical scar	2 (13.3%)	5 (33.3%)	
Infection	4 (26.7%)	2 (13.3%)	
Trauma	3 (20%)	3 (20%)	

**Table 2:** Progression of Vancouver scar scale parameters across treatment sessions

**Parameter Height (cm), Mean±SD**	**Session 1**	**Session 4**	**Session 8**	**Follow-up 2**	**p-value (within group)**
Group F	0.27±0.07	0.15±0.05	0.03±0.03	0.03±0.03	<0.001
Group T	0.32±0.17	0.18±0.12	0.06±0.06	0.05±0.05	<0.001
**Pliability Score, Mean±SD**					
Group F	2.67±0.62	1.80±0.56	1.20±0.41	1.20±0.41	<0.001
Group T	2.73±0.59	1.87±0.52	0.93±0.46	0.80±0.41	<0.001
**Vascularity Score, Mean±SD**					
Group F	1.73±0.46	1.20±0.41	0.93±0.26	0.80±0.41	<0.001
Group T	1.87±0.35	1.60±0.51	0.80±0.41	0.73±0.46	<0.001
**Pigmentation Score, Mean±SD**					
Group F	1.93±0.26	1.93±0.26	1.80±0.41	1.67±0.49	>0.05
Group T	2.00±0.00	2.00±0.00	1.73±0.46	1.73±0.46	>0.05

**Table 3:** Distribution of adverse effects in treatment groups

**Adverse Effect**	**Group F n (%)**	**Group T n (%)**	**p-value**
Telangiectasia	0 (0%)	5 (33.3%)	0.007
Skin atrophy	0 (0%)	3 (20%)	0.033
Superficial ulceration	3 (20%)	1 (6.67%)	0.142
Total complications	3	9	0.127
Patients with any complication	3 (20%)	6 (40%)	0.232
